# Hypoglycemic herbs and their action mechanisms

**DOI:** 10.1186/1749-8546-4-11

**Published:** 2009-06-12

**Authors:** Hongxiang Hui, George Tang, Vay Liang W Go

**Affiliations:** 1 Department of Medicine, Veterans Affairs Greater Los Angeles Healthcare System, Los Angeles, 90073, USA; 2 Division of Medical Genetics, Cedar-Sinai Medical Center, Los Angeles, California 90048, USA; 3 UCLA Center for Excellence in Pancreatic Disease, David Geffen School of Medicine, University of California, Los Angeles, California 90095, USA

## Abstract

Conventional drugs treat diabetes by improving insulin sensitivity, increasing insulin production and/or decreasing the amount of glucose in blood. Several herbal preparations are used to treat diabetes, but their reported hypoglycemic effects are complex or even paradoxical in some cases. This article reviews recent findings about some of the most popular hypoglycemic herbs, such as ginseng, bitter melon and *Coptis chinensis*. Several popular commercially available herbal preparations are also discussed, including ADHF (anti-diabetes herbal formulation), *Jiangtangkeli*, YGD (*Yerbe Mate-Guarana-Damiana*) and BN (*Byakko-ka-ninjin-to*). The efficacy of hypoglycemic herbs is achieved by increasing insulin secretion, enhancing glucose uptake by adipose and muscle tissues, inhibiting glucose absorption from intestine and inhibiting glucose production from heptocytes.

## Background

Diabetes mellitus is a disease in which blood glucose levels are above normal [1]. There are three main types of diabetes, namely type I diabetes (juvenile diabetes), type II diabetes and gestational diabetes. In type I diabetes, the β cells of the pancreas do not make sufficient insulin. Type II diabetes is the major form of diabetes, accounting for approximately 90–95% of all diabetic cases. This form of diabetes usually begins with insulin insensitivity, a condition in which muscle, liver and fat cells do not respond to insulin properly. The pancreas eventually loses the ability to produce and secrete enough insulin in response to food intake. Gestational diabetes is caused by hormonal changes during pregnancy or by insulin insufficiency. Glucose in the blood fails to enter cells, thereby increasing the glucose level in the blood. High blood glucose, also known as hyperglycemia, can damage nerves and blood vessels, leading to complications such as heart disease, stroke, kidney dysfunction, blindness, nerve problems, gum infections and amputation [2]. Insulin injections, glucose-lowering drugs and lifestyle changes, such as exercise, weight control and diet therapy, are recommended for treating diabetes.

Hypoglycemic herbs are widely used as non-prescription treatment for diabetes [3]. However, few herbal medicines have been well characterized and demonstrated the efficacy in systematic clinical trials as those of Western drugs.

This review article highlights the current researches on the efficacy, side effects and action mechanisms of hypoglycemic herbs *in vitro*, *in vivo *and *ex-vivo *systems [4].

### Conventional diabetic drugs

**Figure 1:**
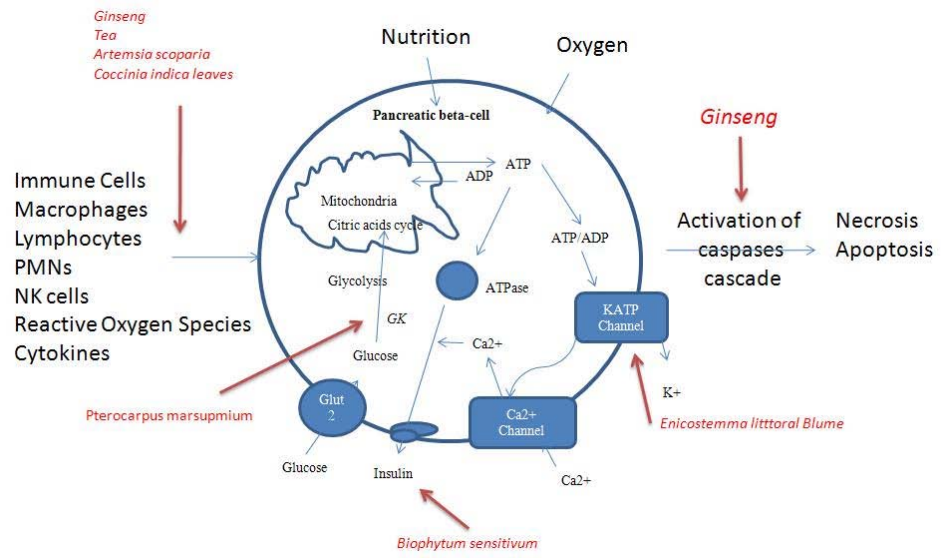
**Insulin secretion and pancreatic-β-cell apoptosis**. Glucose is taken up into β-cells via glucose transporters. It is metabolized in glycolysis and Krebs cycle, resulting in an increased ratio of ATP to ADP in the cytoplasm. This closes ATP-sensitive potassium channels (KATP channels), leading to cell membrane depolarization and subsequently opening voltage-gated Ca2+ channels. These changes increase free Ca2+ concentration ([Ca2+]i) in cytoplasm and eventually triggers insulin secretion. In apoptosis, stimuli promotes the release of caspase activators from mitochondria and result in the activation of caspases procedure, by cleaving the effector caspases, which interacts with a variety of cellular proteins, resulting in directly or indirectly the morphological and biochemical characteristics of cell apoptosis. The action sites of hypoglycemia herbs are indicated with a narrow.

**Figure 2:**
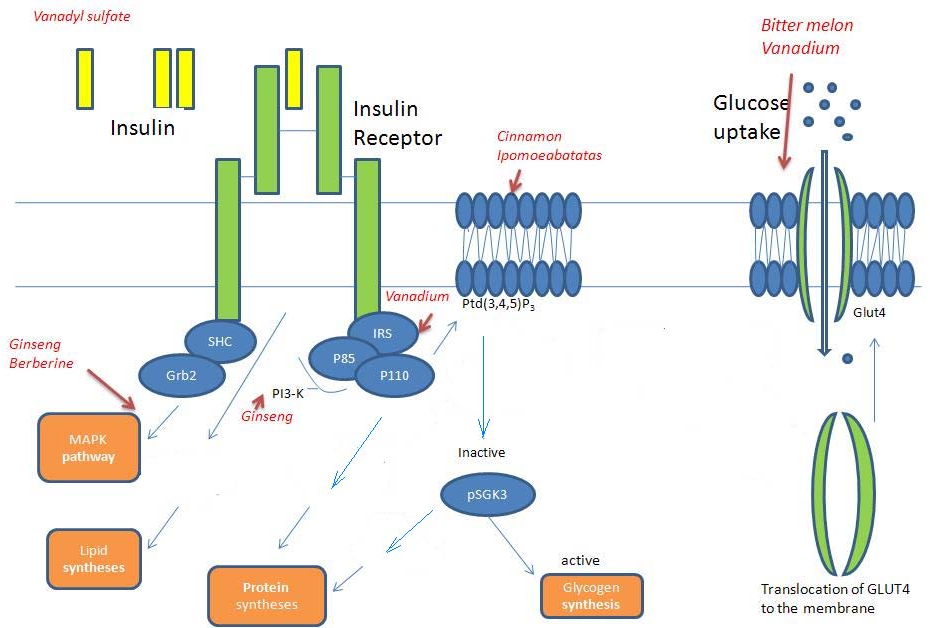
**Insulin signal pathway and insulin insensitive**. The inner part of IR reveals a tyrosine kinase activity and coupled with proteins of Src-homology-collagen-like protein (SHC) and multifunctional docking proteins IRS-1 and IRS-2. The interaction of insulin and IR activates its tyrosine activity and phosphorylates the coupled SHC and subsequently activates, in turn, a series of signal proteins, including the growth factor receptor-binding protein 2 (Grb2), and the ras small guanosine 5'-triphosphate-binding protein. The in turn signaling leads to an activation of the MAPK cascade involved in mitogenesis and the open status of a hexose transporter protein (GLUTs), which is located in the cell membrane and is the only pump to take into glucose for cells. The decreased serine/threonine phosphorylation of IR, inactivates hexokinase and glycogen synthase, as well as defects in the phosphorylation of glucose transporter protein (GLUT4) and genetic primary defect in mitochondrial fatty acid oxidation, leading to insulin resistance and an increase of triglyceride synthesis contribute to this insulin insensitivity. The action sites of hypoglycemia herbs are indicated with an arrow.

Western diabetic drugs correct hypoglycemia by supplementing insulin, improving insulin sensitivity, increasing insulin secretion from the pancreas and/or glucose uptake by tissue cells. Under normal conditions, pancreatic β-cells secrete sufficient insulin to maintain blood glucose concentration within a narrow range (72–126 mg/dL) [5] (Figure 1). The insulin stimulation followed by cascade signaling enhances glucose intake, utilization and storage in various tissues (Figure 2). In diabetic patients, the body loses insulin producing capacity as a result of pancreatic β-cell apoptosis or insulin insensitivity. The cytokines, lipo-toxicity and gluco-toxicity are three major stimuli for β-cell apoptosis [6] (Figure 1).

**Figure 3:**
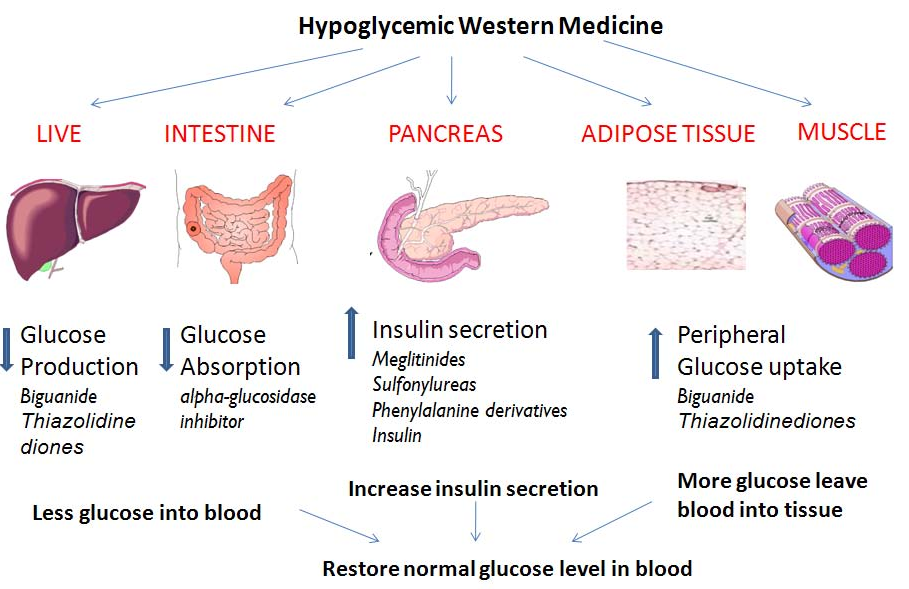
**Action sites of western medicine in diabetes treatment**. Hypoglycemic medicines restore euglycemia via several types, including insulin secretagogues (sulfonylureas, meglitinides), insulin sensitizers (biguanides, metformin, thiazolidinediones), alpha-glucosidase inhibitors (miglitol, acarbose).

There are several types of glucose-lowering drugs [7] (Figure 3), including insulin secretagogues (sulfonylureas, meglitinides), insulin sensitizers (biguanides, metformin, thiazolidinediones), α-glucosidase inhibitors (miglitol, acarbose). New peptide analogs, such as exenatide, liraglutide and DPP-4 inhibitors, increase GLP-1 serum concentration and slow down the gastric emptying [8,9]. Most glucose-lowering drugs, however, may have side effects, such as severe hypoglycemia, lactic acidosis, idiosyncratic liver cell injury, permanent neurological deficit, digestive discomfort, headache, dizziness and even death [10].

### Anti-diabetes herbs

Certain herbs may lower blood glucose [3,11]; however, their test results are subject to several factors. Firstly, each herb contains thousands of components, only a few of which may be therapeutically effective [12]. Secondly, different parts of an herb have different ingredient profiles. Moreover, different extraction methods may yield different active ingredients [13]. Thirdly, herbal formulae containing multiple herbs may have synergistic effects [14,15].

#### Ginseng

The therapeutic potency of ginseng mainly relies on its geographical locality, dosage, processing and types of diabetes. *Panax ginseng *(Chinese or Korean ginseng) has the highest therapeutic potency. *Panax quinquefolius *(American ginseng) is the medium potency grade ginseng, while *Panax japonicus *(Japanese ginseng) is considered the low potency grade ginseng. Thus, the most commonly used therapeutic ginseng is *Panax ginseng*. The anti-tumor, angiomodulating and steroid-like activities of ginseng have been recently delineated [16].

The anti-diabetic effects of ginseng have been investigated with aqueous or ethanol ginseng extracts. A proposed action mechanism has been tested on various animal models [17]. Korean red ginseng (0.1–1.0 g/ml) significantly stimulated insulin release from isolated rat pancreatic islets at 3.3 mM glucose concentration [18]. The treatment with oral administration of H-AG (heat-processed American ginseng) at a dose of 100 mg/kg of body weight for 20 days decreased serum levels of glucose and glycosylated proteins and hemoglobin A1C in streptozotocin (STZ)-induced diabetic rats. The treatment also improved the decreased creatinine clearance level and decreased the accumulation of N (ε)-(carboxymethyl) lysine and its receptors for advanced glycation end product (AGE) expressions in kidney [19]. *Radix Ginseng Alba *improved hyperglycemia in KKAy mice, possibly by blocking intestinal glucose absorption and inhibiting hepatic glucose-6-phosphatase, while *Radix Ginseng Palva *has a similar effect through the up-regulation of adipocytic PPAR-y protein expression and inhibition of intestinal glucose absorption [20].

The treatment of the C57BL/Ks *db/db *mice with *Panax ginseng *berry extract (150 mg/kg of body weight) significantly lowered the fasting blood glucose levels on day 5 and achieved euglycemia on day 12 [21]. Berry extract showed marked anti-obesity effect in obese *ob/ob *and *db/db *mice [22]. Red ginseng lowered hemoglobin A1C to normal range and improved insulin sensitivity [21]. Similarly, extract of American ginseng berry also lowered fasting blood glucose levels significantly in diabetic *ob/ob *mice receiving daily berry juice at 0.6 ml/kg. This hypoglycemic effect continued for at least ten days after the treatment. In addition, reduction of body weight was also observed [23].

While both ginseng root and berry possess anti-diabetic effects [24], ginseng berry seems to be more potent in anti-hyperglycemic activity [25]. Furthermore, only ginseng berry showed marked anti-obesity effects in *ob/ob *mice [24,26].

A total of 705 components have been isolated from ginseng, such as ginsenosides, polysaccharides, peptides and polyacetylenic alcohols, among which ginsenosides are believed to be responsible for ginseng's efficacy [27]. Pharmacological sequential trials of three components, i.e. (1) fat-soluble components, (2) ginseng saponins and (3) a third component with hypoglycemic activity identified the most active components (100-fold more effective than the original water-soluble extract of the ginseng root). Ginseng's clinical efficacy is thought to be medicated by multiple factors [27,28]: the component panaxans (panaxans A to E) elicits hypoglycemia in both normal and diabetic mice; the component adenosine inhibits catecholamine-induced lipolysis; both components of carboxylic acid and peptide 1400 inhibit catecholamine-induced lipolysis in rat epididymal fat pads; and the component DPG-3-2 provokes insulin secretion in diabetic and glucose-loaded normal mice [29]. EPG-3-2, a fraction related to DPG-3-2, also exhibits an anti-lipolytic activity related to anti-obesity effects. Ginsenoside Rg3 inhibits adipocyte differentiation via PPAR-γ pathway in rosiglitazone-treated cells and activates AMPK, a pathway involved in the control of nutritional and hormonal modulation [30]. Ginsenoside Rh2 improves insulin sensitivity in rats fed with fructose rich chow [31]. Therefore, we suggest that the whole extract of ginseng contains multiple biologically active components that stimulate insulin secretion, blocking intestinal glucose absorption and enhancing glucose peripheral utilization.

Ginseng treatment for type II diabetes has been tested in both animal models and human clinical trials. *Panax quinquefolius *(10 g/1 kg diet) increases body weight and decreases cholesterol levels, PPAR actions and triglyceride metabolism in male Zucker diabetic fatty (ZDF) rats [32]. In human clinical trials, *Panax quinquefolius *improves post-prandial glycemia in type II diabetic patients [33]. Single intravenous injection of ginsenoside Rh2 decreases plasma glucose concentrations within 60 minutes in a dose-dependent manner in rats fed with fructose rich chow and STZ-induced insulin resistant rats [30]. A possible mechanism is that ginsenoside Rh2 promotes the release of ACh from nerve terminals which stimulate muscarinic M (3) receptors in pancreatic cells to increase insulin secretion [34].

Ginseng is also used to treat type I diabetic patients. Ginsenosides at 0.1–1.0 g/mL inhibited cytokine-induced apoptosis of β-cells. The action mechanism involves the reduction of nitric oxide (NO), production of reactive oxygen species (ROS) [35], inhibition on p53/p21 expression and inhibition on cleavage of caspases and poly (ADP-ribose) polymerase (PARP) [36].

Not only does ginseng benefit serum glucose control in diabetic patients, but also aids central nervous system complications in them. Alternation expression of NOS gene is implicated in the pathogenesis of numerous secondary complications in diabetic patients. In animal models, enhanced NOS expression was detected in the hippocampus of diabetic rats and the administration of ginseng root suppressed NOS expression [33]. Pharmacological studies confirmed that ginseng possesses multiple actions (central nervous system, neuroprotective, immunomodulation and anticancer effects). Ginsenosides have antioxidant, anti-inflammatory, anti-apoptotic and immuno-stimulant properties [36].

Side-effects of ginseng include insomnia, diarrhea, vaginal bleeding, breast pain, severe headache, schizophrenia and fatal Stevens-Johnson syndrome [37]. The recommended dosage of ginseng application is 1–3 g of root or 200–600 mg of extract [38]. Ginseng has the potential to prolong bleeding time and therefore should not be used concomitantly with warfarin. Moreover, ginseng may cause headache, tremulousness, and manic episodes in patients treated with phenelzine sulfate [39]. Ginseng may interfere with the actions of estrogens or corticosteroids and may impede digoxin metabolism or digoxin monitoring [40].

#### Momordica charantia (bitter melon)

Hypoglycemic effects of bitter melon were demonstrated in cell culture, animal models [41] and human studies [42]. The anti-diabetic components in bitter melon include charantin, vicine, polypeptide-p, alkaloids and other non-specific bioactive components such as anti-oxidants. The major compounds in bitter melon methanol extract, including 5-β, 19-epoxy-3-β, 25-dihydroxycucurbita-6,23(E)-diene (4) and 3-β,7-β,25-trihydroxycucurbita-5,23(E)-dien-19-al (5) showed hypoglycemic effects in the diabetic male *ddY *mice at 400 mg/kg [43]. Oleanolic acid glycosides, compounds from bitter melon, improved glucose tolerance in Type II diabetics by preventing sugar from being absorbed into intestines. Saponin fraction (SF) extracted from bitter melon with PEG/salt aqueous two-phase systems showed hypoglycemic activity in alloxan-induced hyperglycemic mice [44]. Bitter melon increased the mass of β cells in the pancreas and insulin production [45,46]. With edible portion of bitter melon at 10% level in the diet STZ-induced diabetic rats, an amelioration of about 30% in fasting blood glucose was observed [45].

Biochemical studies indicated that bitter melon regulated cell signaling pathways in pancreatic β-cell, adipocytes and muscles. Ethyl acetate (EA) extract of bitter melon activates peroxisome proliferator receptors (PPARs) α and γ [46,47], modulates the phosphorylation of IR and its downstream signaling pathway, thereby lowering plasma apoB-100 and apoB-48 in mice fed with high-fat diet HFD. The momordicosides (Q, R, S and T) stimulate GLUT4 translocation of the cell membrane and increase the activity of AMP-activated protein kinase (AMPK) in both L6 myotubes and 3T3-L1 adipocytes, thereby enhancing fatty acid oxidation and glucose disposal during glucose tolerance tests in both insulin-sensitive and insulin-insensitive mice [48].

Bitter melon can be used as a dietary supplement herbal medicine for the management of diabetes and/or metabolic syndromes [49]. Reported adverse effects of bitter melon include hypoglycemic coma, convulsions in children, reduced fertility in mice, a favism-like syndrome, increased enzyme activities of γ-glutamyl transferase and alkaline phosphotase in animals and headaches in humans. Bitter melon has an additive effect with other glucose-lowering agents [50]. Bitter melon also reduces adiposity in rats fed with HF diet [51].

#### Coptis chinensis (Huanglian)

*Coptis chinensis *is commonly used to treat diabetes in China. Found in plant roots, rhizomes, stems and barks, berberine is an isoquinoline alkaloids and the active ingredient of *Coptis chinensis*.

Intragastric administration of berberine (100 and 200 mg/kg) in diabetic rats decreased fasting blood glucose levels and serum content of TC, TG, LDL-c, increased HDL-c and NO level, and blocked the increase of SOD and GSH-px levels [52,53]. Multiple mechanisms may be responsible for weight reduction and increased insulin response induced by berberine. Glucose's uptake by adipocytes is enhanced by berberine via GLUT1, adenosine monophosphate-activated protein kinase and acetyl-coenzyme A carboxylase phosphorylation [54]. Berberine also increases the PPAR α/δ/γ protein expression in liver [55], increases insulin receptor expression in liver and skeletal muscle cells and improves cellular glucose consumption in the presence of insulin [56]. Berberine increases GLUT4 translocation in adipocytes and myotubes [57], increases AMPK activity, decreases glucose-stimulated insulin secretion (GSIS) and palmitate-potential insulin secretion in MIN6 cells and rat islets [58]. Furthermore, berberine decreases significantly the enzyme activity of intestinal disaccharidases and β-glucuronidase in STZ-induced diabetic rats [59]. Recently, dihydroberberine (dhBBR), an identified BBR berberine derivative, demonstrated *in vivo *beneficial effects in rodents fed with high-fat [60].

Berberine may also relieve some diabetic complications. Studies showed that berberine restored damaged pancreas tissues in diabetic rats induced by alloxan [61]. Berberine ameliorates renal dysfunction in rats with diabetic nephropathy through controlling blood glucose, reduction of oxidative stress and suppressing the polyol pathway [61]. Berberine ameliorates renal injury in STZ-induced diabetes, not by suppression in both oxidative stress and aldose reductase activities [61].

As berberine is an oral hypoglycemic agent in clinical studies, the hypoglycemic effect of berberine was similar to that of metformin in 36 adult patients of recently diagnosed type II diabetes [62]. Berberine also lowered fasting blood glucose and postprandial blood glucose in 48 adult patients of poorly controlled type II diabetes during a 3-month period [62]. In the same trials, the fasting plasma insulin, insulin insensitivity index, the total cholesterol and low-density lipoprotein cholesterol reduced significantly [62].

### Chinese herbal preparations for diabetes

#### ADHF (anti-diabetes herbal formulation)

ADHF was studied in diet-induced type II diabetic animals (C57BL/6J mouse model). The blood glucose level dropped markedly in the mice fed with a diet containing 4% or 8% ADHF. Other diabetic parameters such as insulin insensitivity, histopathological changes in the pancreas and liver were also improved significantly in the mice fed with ADHF [63].

#### Jiangtangkli

*Jiangtangkli*, a Chinese medicine formulation containing *Radix Ginseng *(*Renshen*), improves insulin insensitivity by modulating muscle fiber composition and TNF-α in skeletal muscles in hypertensive and insulin-insensitive fructose-fed rats [64].

#### YGD (Yerbe Mate-Guarana-Damiana)

YGD contains Yerbe Mate (leaves of *Ilex paraguayenis*), Guarana (seeds of *Paullinia cupana*) and Damiana (leaves of *Turnera diffusa*). The YGD capsule delayed the gastric emptying significantly, and increased the time to feel gastric fullness and reduced body weight significantly over 45 days on over-weighted patients treated in a primary health care context.

#### BN (Byakko-ka-ninjin-to)

BN contains *Radix Ginseng *(*Renshen*), *Rhizoma Anemarrhena *(*Zhimu*), *Radix Glycyrrhizae Uralensis *(*Gancao*), gypsum (*Shigao*) and rice. BN lowered blood glucose levels in diabetic mice. Furthermore, ginseng-anemarrhena (or ginseng-licorice) reduced the blood glucose levels more than any individual component did. The study results indicate that the anti-hyperglycemic effect of BN relies on the cooperation of four crude therapeutic components and Ca^2+ ^[65].

**Figure 4:**
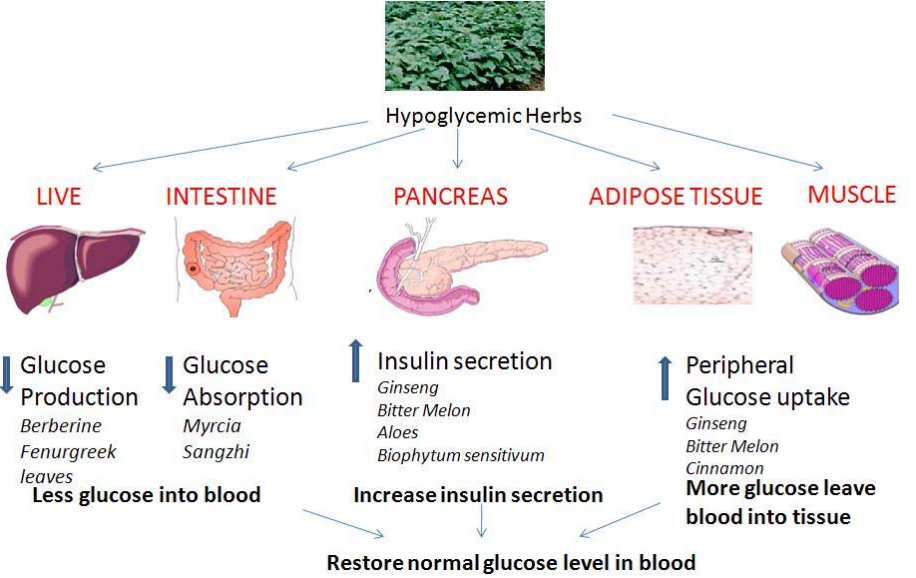
**Action sites of herbs in diabetes treatment**. The efficacy of hypoglycemia herbs has been mediated by increasing insulin secretion (ginseng, bitter melon, aloes, biophytum sensitivum), enhancing glucose uptake by adipose and muscle tissues (ginseng, bitter melon and cinnamon), inhibiting glucose absorption from intestine (myrcia and sanzhi) and inhibiting glucose production from heptocytes (berberine, fenurgreek leaves).

The major goal in treating diabetes is to minimize elevation of blood glucose without causing abnormally low levels of blood glucose. The action mechanisms for hypoglycemic herbs are multiple (Figure 4), such as increasing insulin secretion, enhancing glucose uptake by adipose and muscle tissues, inhibiting glucose absorption from intestine and inhibiting glucose production from heptocytes.

Our literature search [66-99] reveals some commonly used herbs for the management of diabetes mellitus (Table 1).

**Table 1:** Herbs commonly used in diabetes management

Herbs	Components	Anti-diabetic Mechanism	Models of experiments or tests	Application and recommend dosage	Ref
Myrcia	Flavanone glucosides (myrciacitrins) and acetophenone glucosides myrciaphenones)	Inhibit activity of aldose reductase and alpha-glucosidase	Streptozotocin diabetic rats	Type II DM	66
Cinnamon	Cinnulin PF(R)	Improve insulin sensitivity, Decrease fasting blood glucose	Human	Type II DM Type I	67, 68, 69
Enicostemma littorale Blume		Increase the serum insulin through K(+)-ATP channel dependent pathway but did not require Ca2+ influx	Alloxan-induced diabetic rats	Type II DM	70
Biophytum sensitivum		Stimulating the synthesis/release of insulin from the beta cells of Langerhans	Alloxan-induced diabetic rabbits	Type II DM	71
Ipomoea batatas	Caiapo (ipomoea batatas)	Decrease insulin insensitivity, increase adiponectin and decrease fibrinogen levels	Type II diabetic patients	Type II (4 g/d) DM	72, 73
Tithonia diversifolia (Hemsl) A. Gray	Nitobegiku	Reducing insulin insensitivity	KK-Ay-mice	Type II DM	74
Sangzhi	Ramulus mori, SZ	Alpha-glucosidase inhibitory effects	Alloxan induced diabetic rats	Type II DM	75
Galega officinalis		Hypoglycemic effects is independent on a reduction of food intake	ob/ob animals	Type II DM	76
Fenugreek leaves		Similar to glibenclamide, hypoglycemic property and an anti-hyperlipidemic via inferenceiing carbohydrate metabolic enzymes	Streptozotocin induced diabetic rats, human	Type II DM	77, 78
Pterocarpus marsupium		Decrease HK (hexokinase), GK (glucokinase) and PFK (phosphofructokinase)	Human, alloxan-induced diabetic rats	Type II DM	79, 80
Vanadium		Regulate activity of carbohydrate-metabolizing enzymes, and enhance expression of IRS-1 and GLUT4 mRNA in adipocytes	STZ-induced diabetic rats, dexamethasone-induced insulin insensitivity in 3T3-L1 adipocytes	Type II DM	81, 82
Artemisia scoparia	Scoparone (6,7-dimethoxycoumarin	Anti-atherogenic effect; free radical scavenging properties; inhibited iNOS gene expression and inhibited NF-kappaB activation.	Hyperlipidaemic diabetic rabbits, cytokine-induced beta-cell dysfunction	Type I DM, Type II DM	83, 84
Gymnema sylvestre	Gymnemic acids	Controls the activities of phosphorylase, gluconeogenic enzymes and sorbitol dehydrogenase	Alloxan diabetic rabbits	Type II DM complication	85, 86
Daio (Rhei Rhizoma)		Improve kidney function	Patients	Diabetic nephropathy	87
Lupinus termis	Lupinus termis	Regulates acetyl cholinesterase activity, AST (Aspartate aminotransferase), ALT (alanine aminotransferase) and LDH (lactate dehydrogenase)	Alloxan-induced diabetes, patients	Type II DM	88, 89
Tea	EGCG	Reduction of IL-1beta and IFN-gamma-induced nitric oxide (NO) production and levels of NO synthase (iNOS	STZ-treated islets	Type I DM, Type II DM	90, 91
Coccinia indica leaves	Coccinia indica leaf ethanoliextract (CLEt)	Antioxidant property of CLEt	Streptozotocin-diabetic rats	Type II DM	92
Clausena anisata (Willd) Hook [family: Rutaceae]	Terpenoid and coumar	Similar to glibenclamide	Diabetic rats	Type II DM	93
Hovenia dulcis Thunb (HDT)		Similar to glibenclamide, lower blood sugar and hepatic glycogen	Alloxan, induced diabetes rats	Type II DM	94
Aloes		Similar to glibenclamide	Patients, alloxan induced Swiss albino diabetic mice	Type II DM	95, 96
Vanadyl sulfate	bis(maltolato) oxovanadium (IV), BMOV, bis(ethylmaltolato)oxovanadium (IV), BEOV, and bis(isopropylmaltolato)oxovanadium (IV), BIO V,	Insulin-mimetic	Patients, streptozotocin (STZ)-induced type 1 diabetic mice	Type II DM, Type I DM, 100 mg per day	97, 98, 99

### Concerns over herbal treatment for diabetes

While the herbs discussed in this paper have shown efficacy in lowering blood glucose in diabetes patients, the line between whether an herb is a 'drug' or a dietary supplement is unclear. The issues of standardization, characterization, preparation, efficacy and toxicity remain to be addressed.

Herb-drug interaction and herb-herb interaction is another concern. Contrary to some beliefs, herbs can have side-effects. Unfortunately, herb-drug interactions in diabetic treatments have not been well documented. A number of supplements are known to have intrinsic effects on serum glucose, for example, ginseng is hypoglycemic in diabetic patients. Gliclazide is an oral hypoglycemic (anti-diabetic) classified as a sulfonylurea. St John's Wort increases the apparent clearance of gliclazide significantly. Diabetic patients receiving these at the same time should be closely monitored for possible signs of reduced efficacy [100].

## Conclusion

Hypoglycemic herbs are used in Chinese medicine to treat diabetes mellitus. Ginseng, bitter melon and *Coptis chinensis *are used in both types I and II diabetes. The efficacy of hypoglycemic herbs is achieved by increasing insulin secretion, enhancing glucose uptake by adipose and muscle tissues, inhibiting glucose absorption from intestine and inhibiting glucose production from heptocytes.

## Abbreviations

ADP: adenosine diphosphate; AGE: advanced glycation end product; AMPK: AMP-activated protein kinase; ATP: adenosine triphosphate; BUN: blood urea nitrogen; Cr: Creatinine; DPP-4 (DDP IV): dipeptidyl peptidase IV; GLP-1: glucagon-like peptide-1; Grb2: growth factor receptor-binding protein 2; GLUTs: hexose transporter protein; GLUT4: glucose transporter-4; HDL: high-density lipoprotein; HFD: high-fat diet; IRS-1 and IRS-2: insulin receptor substrate-1 and insulin receptor substrate-2; LDL-C: lower-density lipoprotein cholesterol; MRSA: methicillin resistant staphylococcus aureus; NO: nitric oxide; PPAR: peroxisome proliferator receptors; ROS: reactive oxygen species; PARP: poly (ADP-ribose) polymerase; STZ: streptozotocin; SHC: src-homology-collagen-like protein; SOD: superoxide dismutase; TC: total cholesterol; TG: triglyceride; TNF-alpha: tumor necrosis factor alpha; UP24h: urine protein for 24 hours; ZDF: Zucker diabetic fatty rats

## Competing interests

The authors declare that they have no competing interests.

## Authors' contributions

HH conceived and drafted the paper. GT and VLG critically reviewed the literature and revised the manuscript.

## References

[B1] Kempf K, Rathmann W, Herder C (2008). Impaired glucose regulation and type 2 diabetes in children and adolescents. Diabetes Metab Res Rev.

[B2] American Diabetes Association All about diabetes. http://www.diabetes.org/about-diabetes.jsp.

[B3] Yin J, Zhang H, Ye J (2008). Traditional Chinese medicine in treatment of metabolic syndrome. Endocr Metab Immune Disord Drug Targets.

[B4] Xiang YZ, Shang HC, Gao XM, Zhang BL (2008). A comparison of the ancient use of ginseng in traditional Chinese medicine with modern pharmacological experiments and clinical trials. Phytother Res.

[B5] Yu R, Hui H, Shlomo M (2005). Insulin Secretion and Action, Endocrinology (2nd).

[B6] Hui H, Dotta F, Di Mario U, Perfetti R (2004). Role of caspases in the regulation of apoptotic pancreatic islet beta-cells death. J Cell Physiol.

[B7] Modi P (2007). Diabetes beyond insulin: review of new drugs for treatment of diabetes mellitus. Curr Drug Discov Technol.

[B8] Hui H, Zhao X, Perfetti R (2005). Structure and function studies of glucagon-like peptide-1 (GLP-1): the designing of a novel pharmacological agent for the treatment of diabetes. Diabetes Metab Res Rev.

[B9] Garber AJ, Spann SJ (2008). An overview of incretin clinical trials. J Fam Pract.

[B10] Neustadt J, Pieczenik SR (2008). Medication-induced mitochondrial damage and disease. Mol Nutr Food Res.

[B11] Kuriyan R, Rajendran R, Bantwal G, Kurpad AV (2008). Effect of supplementation of Coccinia cordifolia extract on newly detected diabetic patients. Diabetes Care.

[B12] Angelova N, Kong HW, Heijden R van der, Yang SY, Choi YH, Kim HK, Wang M, Hankemeier, Greef J van der, Xu G, Verpoorte R (2008). Recent methodology in the phytochemical analysis of ginseng. Phytochem Anal.

[B13] Shan JJ, Rodgers K, Lai CT, Sutherland SK (2007). Challenges in natural health product research: The importance of standardization. Proc West Pharmacol Soc.

[B14] Liu RH (2004). Potential synergy of phytochemicals in cancer prevention: mechanism of action. J Nutr.

[B15] Kawase M, Wang R, Shiomi T, Saijo R, Yagi K (2000). Antioxidative activity of (-)-epigallocatechin-3-(3"-O-methyl)gallate isolated from fresh tea leaf and preliminary results on its biological activity. Biosci Biotechnol Biochem.

[B16] Yue PY, Mak NK, Cheng YK, Leung KW, Ng TB, Fan DTP, Yeung HW, Wong RNS (2007). Pharmacogenomics and the Yin/Yang actions of ginseng: anti-tumor, angiomodulating and steroid-like activities of ginsenosides. Chin Med.

[B17] Kang KS, Yamabe N, Kim HY, Park JH, Yokozawa T (2008). Therapeutic potential of 20(S)-ginsenoside Rg(3) against streptozotocin-induced diabetic renal damage in rats. Eur J Pharmacol.

[B18] Kim K, Kim HY (2008). Korean red ginseng stimulates insulin release from isolated rat pancreatic islets. J Ethnopharmacol.

[B19] Kim HY, Kang KS, Yamabe N, Nagai R, Yokozawa T (2007). Protective effect of heat-processed American ginseng against diabetic renal damage in rats. J Agric Food Chem.

[B20] Chung SH, Choi CG, Park SH (2001). Comparisons between white ginseng radix and rootlet for antidiabetic activity and mechanism in KKAy mice. Arch Pharm Res.

[B21] Vuksan V, Sung MK, Sievenpiper JL, Stavro PM, Jenkins AL, Di Buono M, Lee KS, Leiter LA, Nam KY, Arnason JT, Choi M, Naeem A (2008). Korean red ginseng (Panax ginseng) improves glucose and insulin regulation in well-controlled, type 2 diabetes: results of a randomized, double-blind, placebo-controlled study of efficacy and safety. Nutr Metab Cardiovasc Dis.

[B22] Xie JT, Aung HH, Wu JA, Attel AS, Yuan CS (2002). Effects of American ginseng berry extract on blood glucose levels in ob/ob mice. Am J Chin Med.

[B23] Xie JT, Wang CZ, Ni M, Wu JA, Mehendale SR, Aung HH, Foo A, Yuan CS (2007). American ginseng berry juice intake reduces blood glucose and body weight in ob/ob mice. J Food Sci.

[B24] Dey L, Xie JT, Wang A, Wu J, Maleckar SA, Yuan CS (2003). Anti-hyperglycemic effects of ginseng: comparison between root and berry. Phytomedicine.

[B25] Dey L, Attele AS, Yuan CS (2002). Alternative therapies for type 2 diabetes. Altern Med Rev.

[B26] Vogler BK, Pittler MH, Ernst E (1999). The efficacy of ginseng: A systematic review of randomized clinical trials. Eur J Clin Pharmacol.

[B27] Kimura M, Kimura I, Chem FJ (1996). Combined potentiating effect of byakko-ka-ninjin-to, its constituents, rhizomes of Anemarrhena asphodeloides, tomosaponin A-III, and calcium on pilocarpine-induced saliva secretion in streptozocin-diabetic mice. Biol Pharm Bull.

[B28] Ng TB, Yeung HW (1985). Hypoglycemic constituents of Panax ginseng. Gen Pharmacol.

[B29] Waki I, Kyo H, Yasuda M, Kimura M (1982). Effects of a hypoglycemic component of ginseng radix on insulin biosynthesis in normal and diabetic animals. J Pharmacobiodyn.

[B30] Hwang JT, Lee MS, Kim HJ, Sung MJ, Kim HY, Kim MS, Kwon DY (2009). Antiobesity effect of ginsenoside Rg3 involves the AMPK and PPAR-gamma signal pathways. Phytother Res.

[B31] Lee WK, Kao ST, Liu IM, Cheng JT (2007). Ginsenoside Rh2 is one of the active principles of Panax ginseng root to improve insulin sensitivity in fructose-rich chow-fed rats. Horm Metab Res.

[B32] Banz WJ, Iqbal MJ, Bollaert M, Chickris N, James B (2007). Higginbotham DA, Peterson R, Murphy L: Ginseng modifies the diabetic phenotype and genes associated with diabetes in the male ZDF rat. Phytomedicine.

[B33] Wu Z, Luo JZ, Luo L (2007). American ginseng modulates pancreatic beta cell activities. Chin Med.

[B34] Lee WK, Kao ST, Liu IM, Cheng JT (2006). Increase of insulin secretion by ginsenoside Rh2 to lower plasma glucose in Wistar rats. Clin Exp Pharmacol Physiol.

[B35] Kim HY, Kim K (2007). Protective effect of ginseng on cytokine-induced apoptosis in pancreatic beta-cells. J Agric Food Chem.

[B36] Xiang YZ, Shang HC, Gao XM, Zhang BL (2008). A comparison of the ancient use of ginseng in traditional Chinese medicine with modern pharmacological experiments and clinical trials. Phytother Res.

[B37] Kiefer D, Pantuso T (2003). Panax ginseng. Am Fam Physician.

[B38] Vuksan V, Sievenpiper JL, Koo VY, Francis T, Beljan-Zdravkovic U, Xu Z, Vidgen E (2000). Related Articles American ginseng (Panax quinquefolius L) reduces postprandial glycemia in nondiabetic subjects and subjects with type 2 diabetes mellitus. Arch Intern Med.

[B39] Abd El Sattar El Batran S, El-Gengaihi SE, El Shabrawy OA (2006). Some toxicological studies of Momordica charantia L. on albino rats in normal and alloxan diabetic rats. J Ethnopharmacol.

[B40] Miller LG (1998). Herbal medicinals: selected clinical considerations focusing on known or potential drug-herb interactions. Arch Intern Med.

[B41] McCarty MF (2004). Does bitter melon contain an activator of AMP-activated kinase?. Med Hypotheses.

[B42] Krawinkel MB, Keding GB (2006). Bitter gourd (Momordica Charantia): A dietary approach to hyperglycemia. Nutr Rev.

[B43] Harinantenaina L, Tanaka M, Takaoka S, Oda M, Mogami O, Uchida M, Asakawa Y (2006). Momordica charantia constituents and antidiabetic screening of the isolated major compounds. Chem Pharm Bull (Tokyo).

[B44] Han C, Hui Q, Wang Y (2008). Hypoglycaemic activity of saponin fraction extracted from Momordica charantia in PEG/salt aqueous two-phase systems. Nat Prod Res.

[B45] Shetty AK, Kumar GS, Sambaiah K, Salimath PV (2005). Effect of bitter gourd (Momordica charantia) on glycaemic status in streptozotocin induced diabetic rats. Plant Foods Hum Nutr.

[B46] Chao CY, Huang C (2003). Bitter Gourd (Momordica charantia) Extract Activates Peroxisome Proliferator-Activated Receptors and Upregulates the Expression of the Acyl CoA Oxidase Gene in H4IIEC3 Hepatoma Cells. J Biomed Sci.

[B47] Chuang CY, Hsu C, Chao CY, Wein YS, Kuo YH, Huang CJ (2006). Fractionation and identification of 9c, 11t, 13t-conjugated linolenic acid as an activator of PPARalpha in bitter gourd (Momordica charantia L). J Biomed Sci.

[B48] Tan MJ, Ye JM, Turner N, Hohnen-Behrens C, Ke CQ, Tang CP, Chen T, Weiss HC, Gesing ER, Rowland A, James DE, Ye Y (2008). Antidiabetic activities of triterpenoids isolated from bitter melon associated with activation of the AMPK pathway. Chem Biol.

[B49] Cefalu WT, Ye J, Wang ZQ (2008). Efficacy of dietary supplementation with botanicals on carbohydrate metabolism in humans. Endocr Metab Immune Disord Drug Targets.

[B50] Basch E, Gabardi S, Ulbricht C (2003). Bitter melon (Momordica charantia): a review of efficacy and safety. Am J Health Syst Pharm.

[B51] Chen Q, Chan LL, Li ET (2003). Bitter melon (Momordica charantia) reduces adiposity, lowers serum insulin and normalizes glucose tolerance in rats fed a high fat diet. J Nutr.

[B52] Yu HH, Kim KJ, Cha JD (2005). Antimicrobial activity of berberine alone and in combination with ampicillin or oxacillin against methicillin-resistant Staphylococcus aureus. J Med Food.

[B53] Tang LQ, Wei W, Chen LM, Liu S (2006). Effects of berberine on diabetes induced by alloxan and a high-fat/high-cholesterol diet in rats. J Ethnopharmacol.

[B54] Zhou L, Yang Y, Wang X, Liu S, Shang W, Yuan G, Li F, Tang J, Chen M, Chen J (2007). Berberine stimulates glucose transport through a mechanism distinct from insulin. Metabolism.

[B55] Zhou JY, Zhou SW, Zhang KB, Tang JL, Guang LX, Ying Y, Xu Y, Zhang L, Li DD (2008). Chronic effects of berberine on blood, liver glucolipid metabolism and liver PPARs expression in diabetic hyperlipidemic rats. Biol Pharm Bull.

[B56] Kong WJ, Zhang H, Song DQ, Xue R, Zhao W, Wei J, Wang YM, Shan N, Zhou ZX, Yang P, You XF, Li ZR, Si SY, Zhao LX, Pan HN, Jiang JD (2009). Berberine reduces insulin resistance through protein kinase C-dependent up-regulation of insulin receptor expression. Metabolism.

[B57] Lee YS, Kim WS, Kim KH, Yoon MJ, Cho HJ, Shen Y, Ye JM, Lee CH, Oh WK, Kim CT, Hohnen-Behrens C, Gosby A, Kraegen EW, James DE, Kim JB (2006). Berberine, a natural plant product, activates AMP-activated protein kinase with beneficial metabolic effects in diabetic and insulin-resistant states. Diabetes.

[B58] Zhou L, Wang X, Shao L, Yang Y, Shang W, Yuan G, Jiang B, Li F, Tang J, Jing H, Chen M (2008). Berberine acutely inhibits insulin secretion from beta-cells through 3', 5'-cyclic adenosine 5'-monophosphate signaling pathway. Endocrinology.

[B59] Liu WH, Hei ZQ, Nie H, Tang FT, Huang HQ, Li XJ, Deng YH, Chen SR, Guo FF, Huang WG, Chen FY, Liu PQ (2008). Berberine ameliorates renal injury in streptozotocin-induced diabetic rats by suppression of both oxidative stress and aldose reductase. Chin Med J (Engl).

[B60] Turner N, Li JY, Gosby A, To SW, Cheng Z, Miyoshi H, Taketo MM, Cooney GJ, Kraegen EW, James DE, Hu LH, Li J, Ye JM (2008). Berberine and its more biologically available derivative, dihydroberberine, inhibit mitochondrial respiratory complex I: a mechanism for the action of berberine to activate AMP-activated protein kinase and improve insulin action. Diabetes.

[B61] Liu WH, Hei ZQ, Nie H, Tang FT, Huang HQ, Li XJ, Deng YH, Chen SR, Guo FF, Huang WG, Chen FY, Liu PQ (2008). Berberine ameliorates renal injury in streptozotocin-induced diabetic rats by suppression of both oxidative stress and aldose reductase. Chin Med J (Engl).

[B62] Yin J, Xing H, Ye J (2008). Efficacy of berberine in patients with type 2 diabetes mellitus. Metabolism.

[B63] Winters WD, Huo YS, Yao DL (2003). Inhibition of the progression of type 2 diabetes in the C57BL/6J mouse model by an anti-diabetes herbal formula. Phytother Res.

[B64] Wang L, Higashiura K, Ura N, Miura T, Shimamoto K (2003). Chinese medicine, Jiang-Tang-Ke-Li, improves insulin resistance by modulating muscle fiber composition and muscle tumor necrosis factor-alpha in fructose-fed rats. Hypertens Res.

[B65] Kimura I, Nakashima N, Sugihara Y, Fu-jun C, Kimura M (1999). The antihyperglycaemic blend effect of traditional chinese medicine byakko-ka-ninjin-to on alloxan and diabetic KK-CA(y) mice. Phytother Res.

[B66] Yoshikawa M, Shimada H, Nishida N, Li Y, Toguchida I, Yamahara J, Matsuda H (1998). Antidiabetic principles of natural medicines. II. Aldose reductase and alpha-glucosidase inhibitors from Brazilian natural medicine, the leaves of Myrcia multiflora DC. (Myrtaceae): structures of myrciacitrins I and II and myrciaphenones A and B. Chem Pharm Bull (Tokyo).

[B67] Ziegenfuss TN, Hofheins JE, Mendel RW, Landis J, Anderson RA (2006). Effects of a water-soluble cinnamon extract on body composition and features of the metabolic syndrome in pre-diabetic men and women. J Int Soc Sports Nutr.

[B68] Anderson RA (2008). Chromium and polyphenols from cinnamon improve insulin sensitivity. Proc Nutr Soc.

[B69] Dannemann K, Hecker W, Haberland H, Herbst A, Galler A, Schäfer T, Brähler E, Kiess W, Kapellen TM (2008). Use of complementary and alternative medicine in children with type 1 diabetes mellitus – prevalence, patterns of use, and costs. Pediatr Diabetes.

[B70] Maroo J, Vasu VT, Aalinkeel R, Gupta S (2002). Glucose lowering effect of aqueous extract of Enicostemma littorale Blume in diabetes: a possible mechanism of action. J Ethnopharmacol.

[B71] Puri D (2001). The insulinotropic activity of a Nepalese medicinal plant Biophytum sensitivum: preliminary experimental study. J Ethnopharmacol.

[B72] Ludvik B, Waldhausl W, Prager R, Kautzky-Willer A, Pacini G (2003). Mode of action of ipomoea batatas (Caiapo) in type 2 diabetic patients. Metabolism.

[B73] Ludvik B, Hanefeld M, Pacini G (2008). Improved metabolic control by Ipomoea batatas (Caiapo) is associated with increased adiponectin and decreased fibrinogen levels in type 2 diabetic subjects. Diabetes Obes Metab.

[B74] Miura T, Furuta K, Yasuda A, Iwamoto N, Kato M, Ishihara E, Ishida T, Tanigawa K (2002). Antidiabetic effect of nitobegiku in KK-Ay diabetic mice. Am J Chin Med.

[B75] Ye F, Shen Z, Xie M (2002). Alpha-glucosidase inhibition from a Chinese medical herb (Ramulus mori) in normal and diabetic rats and mice. Phytomedicine.

[B76] Palit P, Furman BL, Gray AI (1999). Novel weight-reducing activity of Galega officinalis in mice. J Pharm Pharmacol.

[B77] Devi BA, Kamalakkannan N, Prince PS (2003). Supplementation of fenugreek leaves to diabetic rats. Effect on carbohydrate metabolic enzymes in diabetic liver and kidney. Phytother Res.

[B78] Basch E, Ulbricht C, Kuo G, Szapary P, Smith M (2003). Therapeutic applications of fenugreek. Altern Med Rev.

[B79] Grover JK, Vats V, Yadav S (2002). Effect of feeding aqueous extract of Pterocarpus marsupium on glycogen content of tissues and the key enzymes of carbohydrate metabolism. Mol Cell Biochem.

[B80] Dhanabal SP, Kokate CK, Ramanathan M, Kumar EP, Suresh B (2006). Hypoglycaemic activity of Pterocarpus marsupium Roxb. Phytother Res.

[B81] Ramachandran B, Kandaswamy M, Narayanan V, Subramanian S (2003). Insulin mimetic effects of macrocyclic binuclear oxovanadium complexes on streptozotocin-induced experimental diabetes in rats. Diabetes Obes Metab.

[B82] Zuo YQ, Liu WP, Niu YF, Tian CF, Xie MJ, Chen XZ, Li L (2008). Bis(alpha-furancarboxylato)oxovanadium(IV) prevents and improves dexamethasone-induced insulin resistance in 3T3-L1 adipocytes. J Pharm Pharmacol.

[B83] Chen YL, Huang HC, Weng YI, Yu YJ, Lee YT (1994). Morphological evidence for the antiatherogenic effect of scoparone in hyperlipidaemic diabetic rabbits. Cardiovasc Res.

[B84] Kim EK, Kwon KB, Lee JH, Park BH, Park JW, Lee HK, Jhee EC, Yang JY (2007). Inhibition of cytokine-mediated nitric oxide synthase expression in rat insulinoma cells by scoparone. Biol Pharm Bull.

[B85] Shanmugasundaram KR, Panneerselvam C, Samudram P, Shanmugasundaram ER (1981). The insulinotropic activity of Gymnema sylvestre, R. Br. An Indian medical herb used in controlling diabetes mellitus. Pharmacol Res Commun.

[B86] Kanetkar P, Singhal R, Kamat M (2007). Gymnema sylvestre: A Memoir. J Clin Biochem Nutr.

[B87] Goto H, Shimada Y, Tanikawa K, Sato S, Hikiami H, Sekiya N, Terasawa K (2003). Clinical evaluation of the effect of daio (rhei rhizoma) on the progression of diabetic nephropathy with overt proteinuria. Am J Chin Med.

[B88] Mansour HA, Newairy AS, Yousef MI, Sheweita SA (2002). Biochemical study on the effects of some Egyptian herbs in alloxan-induced diabetic rats. Toxicology.

[B89] Knecht KT, Nguyen H, Auker AD, Kinder DH (2006). Effects of extracts of lupine seed on blood glucose levels in glucose resistant mice: antihyperglycemic effects of Lupinus albus (white lupine, Egypt) and Lupinus caudatus (tailcup lupine, Mesa Verde National Park). J Herb Pharmacother.

[B90] Kim MJ, Ryu GR, Chung JS, Sim SS, Min do S, Rhie DJ, Yoon SH, Hahn SJ, Kim MS, Jo YH (2003). Protective effects of epicatechin against the toxic effects of streptozotocin on rat pancreatic islets: in vivo and in vitro. Pancreas.

[B91] Bhathena SJ, Velasquez MT (2002). Beneficial role of dietary phytoestrogens in obesity and diabetes. Am J Clin Nutr.

[B92] Venkateswaran S, Pari L (2003). Effect of Coccinia indica leaf extract on plasma antioxidants in streptozotocin-induced experimental diabetes in rats. Phytother Res.

[B93] Ojewole JA (2002). Hypoglycaemic effect of Clausena anisata (Willd) Hook methanolic root extract in rats. J Ethnopharmacol.

[B94] Ji Y, Chen S, Zhang K, Wang W (2002). Effects of Hovenia dulcis Thunb on blood sugar and hepatic glycogen in diabetic mice. Zhongyaocai.

[B95] Ghannam N, Kingston M, Al-Meshaal IA, Tariq M, Parman NS, Woodhouse N (1986). The antidiabetic activity of aloes: preliminary clinical and experimental observations. Horm Res.

[B96] Okyar A, Can A, Akev N, Baktir G, Sütlüpinar N (2001). Effect of Aloe vera leaves on blood glucose level in type I and type II diabetic rat models. Phytother Res.

[B97] Yanardag R, Bolkent S, Karabulut-Bulan O, Tunali S (2003). Effects of vanadyl sulfate on kidney in experimental diabetes. Biol Trace Elem Res.

[B98] Thompson KH, Liboiron BD, Sun Y, Bellman KD, Setyawati IA, Patrick BO, Karunaratne V, Rawji G, Wheeler J, Sutton K, Bhanot S, Cassidy C, McNeill JH, Yuen VG, Orvig C (2003). Preparation and characterization of vanadyl complexes with bidentate maltol-type ligands; in vivo comparisons of anti-diabetic therapeutic potential. J Biol Inorg Chem.

[B99] Karmaker S, Saha TK, Sakurai H (2008). Antidiabetic activity of the orally effective vanadyl-poly (gamma-glutamic acid) complex in streptozotocin(STZ)-induced type 1 diabetic mice. J Biomater Appl.

[B100] Xu H, Williams KM, Liauw WS, Murray M, Day RO, McLachlan AJ (2008). Effects of St John's wort and CYP2C9 genotype on the pharmacokinetics and pharmacodynamics of gliclazide. Br J Pharmacol.

